# Supplementary material for: Clostridioides difficile stimulates CCL20 expression in human colonoid monolayers in a transwell-based co-culture system that supports its anaerobic growth
Source: bioRxiv. 2026 Jul 1:2026.04.28.721417. Originally published 2026 Apr 29. Preprint. [Version 2] doi: 10.64898/2026.04.28.721417 (PMC13142344; doi:10.64898/2026.04.28.721417)
Supplement: Supplement 1 [file media-1.docx]

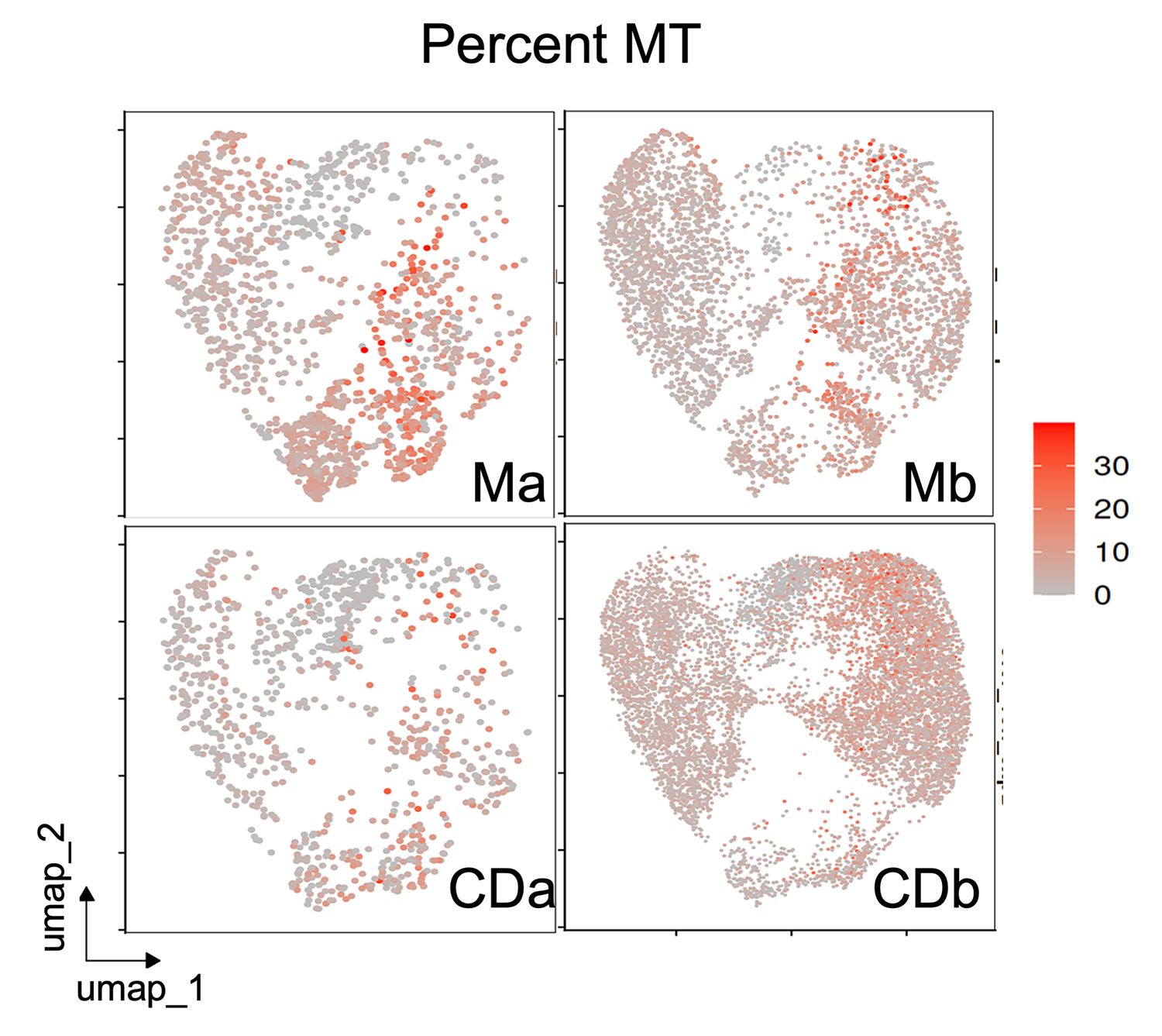


Supplemental Figure S1. Mitochondrial gene expression in samples.

Feature plot indicating percent mitochondrial gene expression (percent MT) in each cell in each sample. Grey indicates low percentage, shades of red indicate higher percentage.
